# Determining solar effects in Neptune's atmosphere

**DOI:** 10.1038/ncomms11976

**Published:** 2016-07-15

**Authors:** K. L. Aplin, R. G. Harrison

**Affiliations:** 1 Department of Physics, University of Oxford, Denys Wilkinson Building, Keble Road, Oxford OX1 3RH, UK; 2 Department of Meteorology, University of Reading, PO Box 243, Earley Gate, Reading RG6 6BB, UK

## Abstract

Long-duration observations of Neptune's brightness at two visible wavelengths provide a disk-averaged estimate of its atmospheric aerosol. Brightness variations were previously associated with the 11-year solar cycle, through solar-modulated mechanisms linked with either ultraviolet or galactic cosmic ray (GCR) effects on atmospheric particles. Here, we use a recently extended brightness data set (1972–2014), with physically realistic modelling to show, rather than alternatives, ultraviolet and GCR are likely to be modulating Neptune's atmosphere in combination. The importance of GCR is further supported by the response of Neptune's atmosphere to an intermittent 1.5- to 1.9-year periodicity, which occurred preferentially in GCR (not ultraviolet) during the mid-1980s. This periodicity was detected both at Earth, and in GCR measured by Voyager 2, then near Neptune. A similar coincident variability in Neptune's brightness suggests nucleation onto GCR ions. Both GCR and ultraviolet mechanisms may occur more rapidly than the subsequent atmospheric particle transport.

## Abstract (web summary)

The causes behind fluctuations in Neptune's brightness as observed from Earth have proved enigmatic. Here, Aplin and Harrison use photometric observations to show that solar ultraviolet radiation and galactic cosmic rays combined are responsible for the fluctuations originating in Neptune's atmosphere.

## Graphical abstract

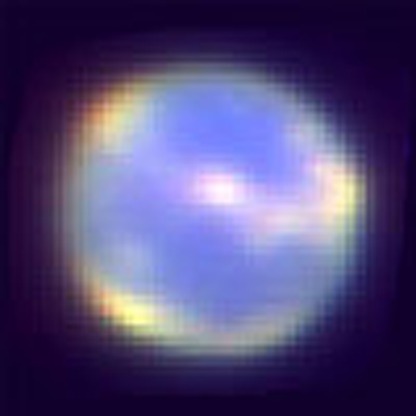

Long-term observations of Neptune from a ground-based telescope show variations in the planet's disk-averaged brightness, which are associated with changes in the reflectivity (albedo) of the planet from its atmospheric aerosol and clouds. Although seasonal variations dominate the time series, Lockwood and Thompson^1^ showed, using data from 1972 to 1996, that small fluctuations in Neptune's brightness at two visible wavelengths followed the 11-year solar cycle. They examined two quantities known to vary closely with solar activity. The first, solar ultraviolet radiation, is linked to photochemical variations in Neptune's atmospheric aerosol particles, and the second, galactic cosmic rays (GCR), may create some of Neptune's aerosol through ion-induced nucleation. It was not possible to discriminate between the ultraviolet and GCR effects, although the relationship with ultraviolet was slightly more statistically robust^1^. The Neptune magnitude-solar activity relationship broke down after 1996 (refs 1, 2), but recent extension of the data^3^ encourages its re-examination. Supporting evidence for a solar cycle in infrared observations from 1975 to 2007 (ref. 4) further motivates a fresh consideration of the origin of the short-term variability in Neptune's albedo.

Both the ultraviolet and GCR mechanisms can, in principle, account for the changes observed in the photometric observations, which originate in Neptune's stratosphere and troposphere^1^,^5^. The ultraviolet mechanism was originally proposed^6^ to explain the solar cycle signal when it was first reported in Neptune's albedo^7^. It was suggested that ultraviolet-triggered surface chemistry on pre-existing aerosol particles varied the optical properties of Neptune's stratospheric aerosol through a darkening in colour (‘tanning'), detectable in the photometric measurements. The GCR-driven mechanism was proposed for Neptune^8^,^9^, through direct (‘Wilson') condensation of supersaturated gas onto atmospheric ions^10^, causing particle growth ultimately detectable at optical wavelengths. The possibility that charge-related effects could modulate the atmospheres of the outer planets, where variations in solar irradiance are proportionately less important^11^, contrasts with the small likely role of energetic particles in Earth's atmosphere^12^ and provides a further motivation for this study.

The two proposed mechanisms for external solar forcing of Neptune are essentially heliospheric (through GCR) or photospheric (through solar ultraviolet) in origin. The analysis to investigate them here uses two approaches. First, the relationships between Neptune's magnitude, solar ultraviolet radiation and GCRs are studied with multiple regression, allowing both the proposed mechanisms to act together. We find that, when the extra degrees of freedom are accounted for, including both ultraviolet and GCR improves prediction of the magnitude fluctuations. Second, we examine the relatively rapid fluctuations apparent during the mid-1980s in the Neptune astronomical data. This enhanced variability coincided with a known episode of quasi-periodic fluctuations present in GCR^13^, centred around 1.68 years. Investigating Neptune's atmospheric variability in the 1.5 to 2 year range therefore presents a method by which to separate the two different suggested solar-modulated influences, an approach previously used to separate coincident terrestrial atmospheric responses^14^.

## Results

### The extended Neptune photometric data set 1972–2014

**Figure 1:**
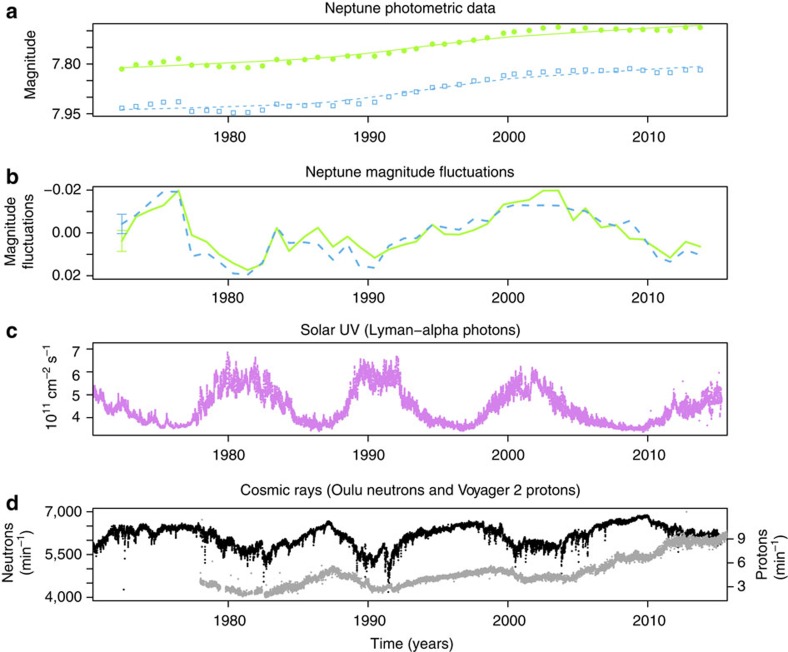
Time series of Neptune's brightness and solar modulated parameters. (**a**) Neptune brightness (astronomical magnitude, where smaller values represent a greater signal) time series at 472 nm (blue squares) and 551 nm (green circles), from ref. 3, each smoothed with a lowess fit (blue dashed line or green solid line). (**b**) Magnitude fluctuations after detrending (**a**) with a lowess fit. The maximum s.e.m. in each data set is shown as a single error bar on the far left. (**c**) Lyman-alpha (ultraviolet) radiation at 121.5 nm. (**d**) Cosmic ray count rate at Earth's surface and in the heliosphere, showing terrestrial neutron monitor data from Oulu, Finland, (black) and Voyager 2 LECP instrument daily mean flux of cosmic ray protons >70 MeV (grey). Data are described in full in the ‘Methods' section.

Regular photometric observations of the magnitude (brightness) of Neptune have been made since 1972, through well-characterised visible bandpass filters of width ∼20 nm centred at 472 nm (‘b', blue) and 551 nm (‘y', green) using a 21-in telescope at Lowell Observatory, Arizona^2^, Fig. 1a. Each magnitude value is typically determined from between 4 and 39 nights of data (median 9 nights), taken around the time the planet is brightest in the sky (opposition)^3^. Standard errors in the magnitude measurements, determined from the standard deviations and number of nights of observation were typically ±0.001 (ref. 3). Sampling intervals varied between 0.7 and 1.2 years, with median interval of 1.04 years. Figure 1 summarizes the data, with Fig. 1a showing the measured magnitudes, Fig 1b showing the magnitude fluctuations, Fig. 1c showing the ultraviolet data and Fig. 1d showing GCR measured both at Earth and in space. More information on the data is given in the ‘Methods' section.

The correlation between the 29 data points and 30-day running means of ultraviolet, sunspots and GCR with a range of lags was calculated previously^1^. The correlation between Neptune's magnitude fluctuations and ultraviolet was statistically significant, whereas the correlation with GCR was not, on which basis it was concluded that ultraviolet was the more likely mechanism^1^. This analysis^1^ did not allow for the possibility that both the physically plausible mechanisms involving ultraviolet and GCR could be acting in combination in Neptune's atmosphere to cause the observed albedo variations. If so, multiple regression may be more appropriate than treating the two proposed mechanisms independently. The earlier analysis^1^ was statistically rather than physically based, whereas we have used standard ion–aerosol theory to guide our statistical approach. Finally, extra data have recently been made available^3^, which we now include.

Previous analyses^1^ assumed linear relationships between ultraviolet (UV) and albedo, and GCR and albedo. Although the ultraviolet-albedo relationship is generally assumed to be linear^6^, this assumption is not necessarily appropriate for the GCR mechanism. In this case, the albedo changes are likely to be proportional to the number of ion-induced particles. The number of particles formed is controlled by the ion-induced nucleation rate^9^, which is proportional to the atmospheric ion (or electron) concentration *n*, where the ions are singly charged, nanometre-sized clusters created by GCR ionization^11^ with volumetric production rate *q*. In ion–aerosol theory, there are two possible limiting regimes linking *n* and *q*. First, the recombination limit of negligible aerosol, in which the ion concentration *n* is controlled by ion–ion or ion–electron self-recombination and 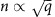. Second, in the attachment regime, the ion concentration is limited by attachment to any pre-existing particles (such as aerosols, haze, clouds or dust) and *n*∝*q* (for example, see ref. 11). Assuming that the GCR count rate provides an estimate of *q* (for example, see ref. 15), a set of possible statistical relationships was investigated of the form:


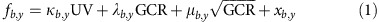


where *f*_b,y_ are the measured magnitude fluctuations in the *b* (472 nm) or *y* (551 nm) wavelength ranges, *κ, λ* and *μ* are coefficients for the *b* or *y* data representing the ultraviolet mechanism, ion attachment and ion recombination, respectively, and *x* is a constant for the *b* or *y* data. Daily Lyman-alpha and Oulu neutron counter (GCR) data, averaged for ±20 days around the observation date given in ref. 3, were used as measurements of ultraviolet (UV) and GCR (see the ‘Methods' section). The regressions were weighted according to the standard errors in the magnitude data described above, and the errors in ultraviolet and GCR data were assumed to be negligible in comparison with those in the magnitude data. As the attachment and recombination regimes cannot act simultaneously at the same location, this set of statistical relationships represents the integrated effect of the different atmospheric layers observed at Earth through each filter.

**Figure 2:**
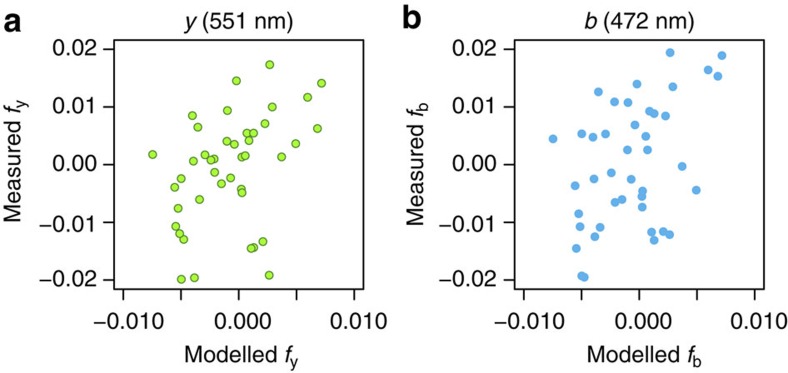
Physically based linear regression models to explain Neptune magnitude fluctuations. The models include ultraviolet radiation, attachment and recombination of GCR-created ions—Case 7 in Table 1—for (**a**) *f*_*y*_ at 551 nm and (**b**) *f*_*b*_ at 472 nm. The coefficients determined in equation (1) for, respectively, (**a**) *κ*=0.010±0.004 cm^2^s, *λ*=(2±1) × 10^−4^ min, *μ*=−0.04±0.02 min^0.5^, *x*=1.5±0.8 mag (where mag is astronomical magnitude), and (**b**) *κ*=0.011±0.005 cm^2^s, *λ*=(2±1.5) × 10^−4^ min, *μ*=−0.03±0.02 min^0.5^ and *x*=1.1±0.9 mag.

The adjusted coefficient of determination (*R*^2^), that is, fraction of the variance explained by the fit, corrected for the number of points and fitted variables, was used to evaluate each model, summarized in Table 1. For the *y* filter, adjusted *R*^2^ was improved to 0.14 (see case 7 in Table 1) when both the ultraviolet and GCR-related mechanisms were included rather than considering the mechanisms separately, which only explained 2–8% of the variance. Considering both GCR-created ionization and ultraviolet therefore permits rejection of the null hypothesis, which is that including both ultraviolet and GCR does not improve the fit (for example, see ref. 16). The improvement from including both GCR and ultraviolet is less marked for the *b* filter data, with the fit between ultraviolet and magnitude fluctuation slightly better than for GCR, ultraviolet and magnitude fluctuation. The measured and modelled magnitude fluctuations are compared in Fig. 2.

One possible explanation for the differences between the *b* and *y* filter responses relates to the different parts of Neptune's atmosphere accessed by each filter. The 442 nm *b* filter responds to stratospheric haze particles, whereas the 551 nm *y* filter is more sensitive to the optical properties of tropospheric aerosols^6^. Ultraviolet will be absorbed by, for example, haze layers^17^ as it passes through the atmosphere, whereas highly energetic secondary GCR, typically muons of several GeV, can readily penetrate the deep atmosphere (for example, see ref. 11), with the maximum ionization, known in the terrestrial atmosphere as the Pfotzer–Regener maximum^15^, expected at about 40 hPa (ref. 9). It is therefore plausible that particles seen by the *b* filter in the haze layers would respond preferentially to ultraviolet, but that the changes in the *y* filter wavelength can be better explained by the inclusion of GCR in the model.

### Spectral analysis

Particle detectors on the Voyager 2 spacecraft measured primary GCR as they passed out of the Solar System. These measurements showed variability on 1 to 2 year timescales^18^, which was at its strongest in primary GCR in the 1980s, consistently with terrestrial neutron monitor data^19^. Although similar variability is apparent in terrestrial GCR (neutron monitor) measurements, it is not in the solar 10.7-cm radio flux, a widely-used index of solar radiative emissions^20^. Beyond GCR data, this variability in the 1980s has also been identified in other heliospherically modulated quantities^19^, such as terrestrial surface atmospheric electricity data^21^. In contrast, such variability is not apparent in photospheric quantities such as solar ultraviolet, which makes this periodicity a useful diagnostic for separating photospheric and heliospherically modulated effects^14^.

**Figure 3:**
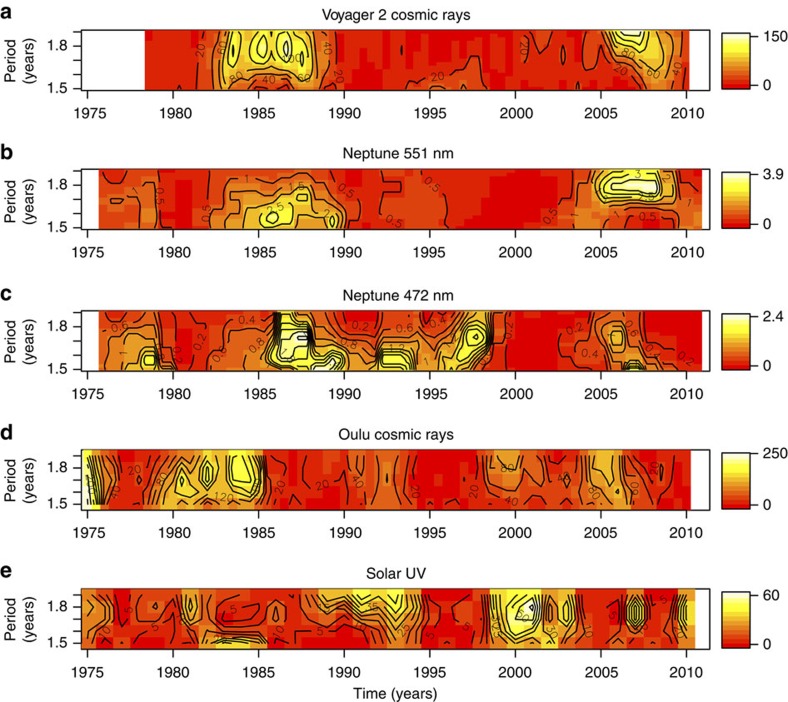
Spectral power densities between 1.5 and 1.9 years. Moving-window spectrograms derive the SPD, normalized by the variance to be dimensionless, for periodicities between 1.5 and 1.9 years from (**a**) the Voyager 2 LECP instrument proton data, (**b**) Neptune's magnitude fluctuations at 551 nm, (**c**) 472 nm, (**d**) Oulu neutron monitor data and (**e**) solar ultraviolet (Lyman-alpha) radiation. Contours of SPD are shown, with colour for added emphasis. The data and spectrogram calculations are described in full in the ‘Methods' section.

To pursue this for Neptune's atmosphere we consider whether the cosmic ray variability observed at Earth is also present at Neptune. After establishing that it is, we then consider when the variability on these timescales occurs in Neptune's atmosphere, GCR and, for completeness, solar ultraviolet. One approach to evaluating variations such as these within specified frequency ranges is to use a periodogram, or a series of periodograms selecting successive time intervals. Using successive periodograms, calculated using the Lomb–Scargle method for irregularly spaced data, the temporal variations of spectral power density (SPD) between 1.5 and 1.9 years in Neptune's magnitude, cosmic rays from both Voyager 2 and Oulu and ultraviolet (Lyman-alpha) radiation are shown in Fig. 3 as a moving-window spectrogram. The cosmic ray data used are protons of energy >70 MeV measured by the Voyager 2 Low Energy Charged Particle (LECP) instrument^22^. Figure 3a presents a spectrogram of the data from Voyager 2, indicating strong spectral power at 1.5–1.9-year periodicities from about 1983 to 1987, when Voyager 2 was travelling from Saturn to Neptune. (Further details of the spectral data processing are given in the ‘Methods' section). Voyager 2's closest approach to Uranus was on 24 January 1986, and to Neptune on 25 August 1989 (ref. 23).

**Figure 4:**
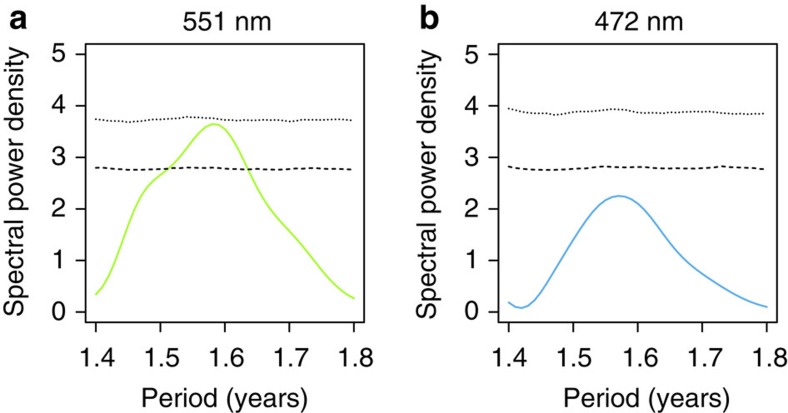
Estimate of statistical significance of the SPD at 1.5–1.9 years during the 1980s. Dimensionless power spectral density (solid lines) calculated for (**a**) 551 nm and (**b**) 472 nm data from 1980 to 1994, using the Lomb–Scargle method after de-trending with a loess fit. The statistical significance of the spectral peaks has been estimated using a Monte-Carlo procedure: dashed and dotted lines show the upper 95th and 99th percentiles of 50,000 realizations of the power spectra calculated in the same way as the spectral peak, but after random shuffling of the magnitude data.

Figure 3a–c shows spectrograms generated from the data collected at Neptune, Fig. 3d shows terrestrial cosmic rays measured at Oulu in Finland and Fig. 3e shows the ultraviolet data. The Oulu and Voyager 2 cosmic ray data support earlier findings^17^, in that the spectral variability appears first in GCR at Earth (Fig. 3d), before reaching Voyager 2 (Fig. 3a) consistent with outward propagation of a heliospheric feature. The Neptune and Voyager 2 spectrograms show similarities in their time evolution, with coincident increased SPD during the 1980s. To evaluate the significance of this enhanced SPD, a Monte-Carlo procedure was used. Using random shuffling of the magnitude fluctuation data, many (50,000) randomised power spectra were obtained; a spectral peak at ∼1.6 years has a probability of being caused by chance of about 1% (*P*<0.02), see Fig. 4.

In contrast, the spectrogram derived from the Lyman-alpha data (Fig. 3e) shows minimal variability in the range of periodicities considered during the mid-1980s. Hence the Voyager 2 cosmic ray data establish that the 1.5–1.9-year periodicity was present both in Neptune's atmosphere and in GCR near Neptune during the 1980s.

The 1.5–1.9-year spectral feature in heliospheric GCR can be used as a ‘fingerprint' of a possible GCR influence in atmospheric properties such as clouds^14^. Comparing the strength of this feature on Neptune with GCR therefore provides a method to separate ultraviolet and GCR effects on Neptune's albedo. However, there may be a lag in Neptune's measured albedo in response to external forcing, due to the internal timescales of particle production and movement in Neptune's atmosphere. These processes were described^6^ as methane being injected into the stratosphere and upper troposphere by convection, where photochemical, nucleation and sedimentation processes act on timescales of typically a few Earth years. The photochemical colour changes postulated to be the ultraviolet mechanism providing solar modulation of the albedo are thought to act on 0.2–2-year timescales^6^. Guided by these ideas, it was found that the fit statistics summarized in Table 1 could be improved by allowing Neptune's albedo to lag ultraviolet and GCR.

**Figure 5:**
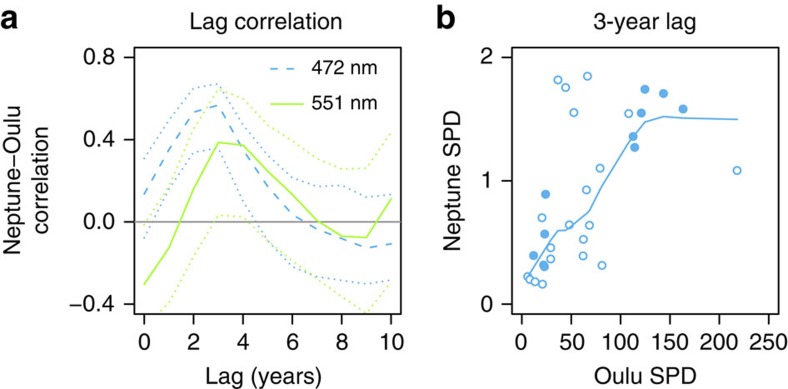
Relationships between 1.5 and 1.9-year spectral power densities for Neptune and GCRs. (**a**) Correlation between mean normalized SPD for periodicities between 1.5 and 1.9 years in Neptune's atmosphere against the same periodicity in GCRs at Oulu, for Neptune lagging Oulu by 0–10 years. Dotted lines mark 95% confidence limits from multiple (10,000) realizations of the SPDs calculated with the uncertainties in the magnitude fluctuations. (**b**) Average Neptune 472 nm SPD against GCR SPD data values for the 1.5–1.9-year periodicity, with Neptune lagging Oulu by 3 years. The filled circles are from 1980 to 1989, when the 1.5–1.9-year periodicity was particularly strong, and the rest of the data are open circles. A lowess fit to all the data points is also shown (solid line).

We have further investigated the delays in Neptune's atmospheric response by carrying out a lag correlation analysis between the average 1.5–1.9-year SPD in GCR, as for Fig. 3, and the same quantity in Neptune's magnitude, Fig. 5a. The peak response is achieved at a lag of 3 years for both wavelengths, and Fig. 5b indicates that, with a 3-year lag, 32% of the variance in the Neptune 472 nm SPD can be explained by GCR at *P*<0.001 confidence (18% for the 551 nm SPD at *P*<0.05 confidence). The calculated lag is robust to errors in the SPD, as determined by recalculating the spectra many (10,000) times, including a normally distributed random error within the quoted uncertainty on the magnitude measurements^3^. Supplementary Fig. 1 is a version of Fig. 5 calculated for the 1.5–1.9-year SPD in Lyman-alpha (ultraviolet) radiation. Unlike the GCR-magnitude relationship in SPD (Fig. 5) which shows an almost linear dependency in the 1980s, there is no statistically significant linear effect between the 482 nm SPD and ultraviolet (or, indeed, for the 551 nm SPD against the ultraviolet SPD, not plotted). This indicates that some of the spectral features previously identified in the GCR data during the 1980s have propagated into Neptune's atmosphere for detection at 472 nm, which is not replicated for the ultraviolet data.

Returning to the GCR effects, and restricting the analysis to data from the 1980s, when the spectral feature was particularly strong and known to be present close to Neptune, then 87% of the variance in this intermittent periodicity at 472 nm can be explained solely by GCR at the *P*<0.001 confidence level. For the *y* data at 551 nm, the *R*^2^ remains 18% during the 1980s.

Estimates using classical cloud physics theory^24^ for plausible parameters of ion-induced particle growth at Neptune (see the ‘Methods' section) indicate that newly nucleated particles could grow to optical wavelengths relatively quickly, with timescales from tens of minutes to hours. This rapid ion growth timescale implies that the lagged GCR effects observed in Neptune's magnitude fluctuations could arise from the propagation of the 1.5–1.9-year periodicity outwards through the heliosphere, suggested by the lag between the spectral features in Voyager 2 (Fig. 3a) and Oulu (Fig. 3d).

The two analyses presented in this paper, by multiple regression and spectral techniques, represent different effects. The multiple regression evaluates the net response of Neptune's magnitude to both GCR and ultraviolet forcing, whereas the SPD approach only addresses the sensitivity of Neptune's atmosphere to one forcing, that of GCR on 1.5–1.9-year timescales. For 472 nm, variability at these periodicities during the 1980s is well explained by GCR. In terms of net response over the whole data set (1972–2014), fluctuations in the 472 nm filter data are most effectively accounted for by ultraviolet variations alone, but at 551 nm there is a combined effect of GCR and ultraviolet.

## Discussion

Two alternative origins have previously been proposed^2^ for the decadal variations observed in Neptune's atmosphere, external forcing (solar modulation) or chance. On the basis of a statistical argument, GCR or ultraviolet presented alternatives as solar forcing agents^1^. Including the most recent data, we now find that considering GCR and ultraviolet as joint contributors to Neptune's atmospheric variability is stronger than an either–or scenario. Our model's explanatory power is enhanced by including realistic ion–aerosol physics, allowing for ion loss both by attachment to aerosol and by ion–ion or ion–electron recombination.

Ion–aerosol theory considerations indicate that, in cloudy or hazy conditions, ions attach to aerosol particles (see equation 1). This has two consequences. First, haze particles or cloud droplets will become charged by ion attachment. The charge does not depend on the ionization rate^25^, so will not generate the photometric variability analysed here. Second, the associated ion depletion will reduce opportunities for ion-induced nucleation. As Neptune's stratosphere and troposphere are rich in haze and cloud particles^5^,^6^, this suggests a role for transport processes^6^,^26^ in moving nucleated particles to regions where they become detectable in the photometric observations.

Our work provides new evidence for solar forcing in Neptune's atmosphere on sub-seasonal timescales, through both ultraviolet-driven and GCR mechanisms. The lags in Neptune's response to external forcing present in both GCR and ultraviolet data over the entire time series, and in the GCR SPD during the 1980s, are consistent both with each other and with known particle movement timescales^6^. Further investigation is needed to understand the potentially very different timescales associated with both particle-modulating mechanisms, and the relevant transport processes within Neptune's atmosphere.

## Methods

### Neptune magnitude data

The Neptune magnitude data has been obtained by Dr W Lockwood and collaborators from the Lowell Observatory, Arizona over many years^2^,^3^. Neptune's magnitude is measured with a 21-in reflector telescope using differential photometry, a technique based on measuring the brightness of a target object relative to comparison stars. The data are filtered in the visible with filters called ‘b' (centred on 472 nm) and ‘y' (centred on 551 nm). Filter-response functions and details of the long-term stability and errors are all given in ref. 2.

**Figure 6:**
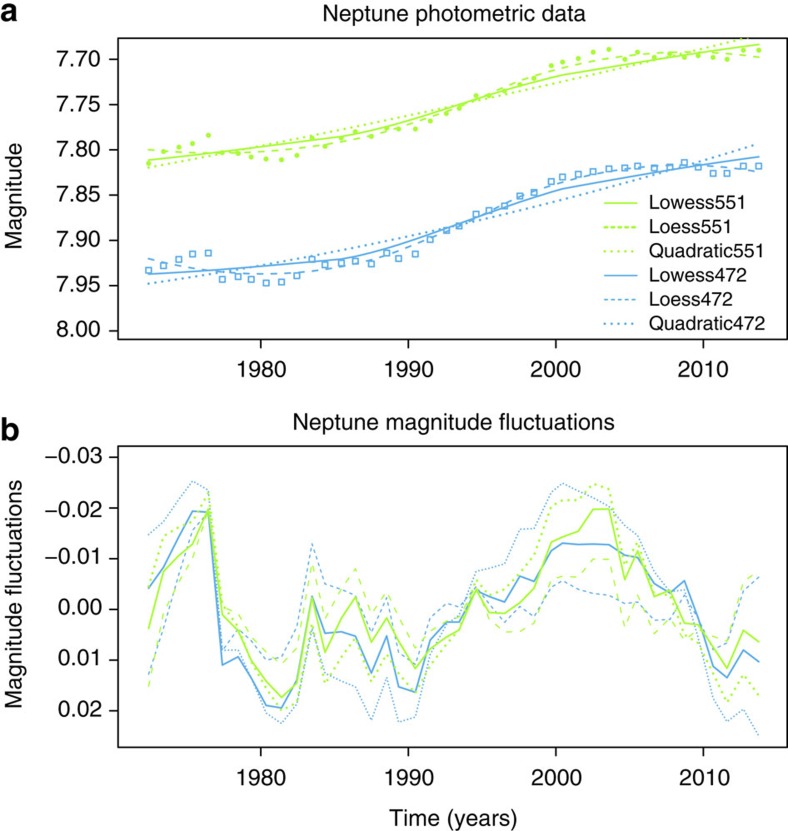
Comparison of smoothing approaches for Neptune magnitude time series data. (**a**) Raw data are shown as points (blue squares for 472 nm, green circles for 551 nm), with three different smoothing lines as indicated on the legend. (**b**) fluctuations calculated using the three different fits, with the same lines as indicated in the legend for **a**.

Detrending the Neptune magnitude data is necessary to remove the slow seasonal related increase in Neptune's brightness (for example, see refs 1, 2, 27). Following the approach in ref. 1, we have applied smoothing curves to the magnitude data to remove the low-frequency seasonal changes and look at fluctuations occurring on more rapid timescales. A quadratic detrend was applied in earlier analyses^1^,^2^, but rather than assume an arbitrary polynomial, we have applied robust local smoothing methods and compared them with the quadratic in Fig. 6a. The lowess^28^ fit and the newer algorithm, loess^29^, are well-established non-parametric local smoothers, weighted towards points near the region to be fitted. It can be seen from Fig. 6b that the key features in the magnitude fluctuations are preserved independently of the smoothing fit chosen.

A Kolmogorov–Smirnov (KS) statistical test has also been used to establish whether the magnitude fluctuations calculated using any of the three smoothing fits differ statistically. Importantly, the Kolmogorov–Smirnov test does not make any assumptions about the distribution of the data (for example, see ref. 30). Table 2 indicates that for most of the types of smoothing used, the calculated magnitude fluctuations are not significantly different.

### Cosmic ray data

GCRs are energetic particles generated outside the Solar System. They are mainly protons, which are most abundant, and alpha particles (helium nuclei)^31^. Cosmic rays ionize atmospheres by colliding with molecules and inducing a cascade of secondary ionizing and non-ionizing particles; they are the major source of ionization in many planetary atmospheres^11^. Neutron monitor measurements of GCR are used here as an indicator of atmospheric ionization. Neutrons are non-ionizing radiation formed by GCR decaying in Earth's atmosphere and are measured by a network of terrestrial monitors, described below. GCR can also be measured directly by spacecraft. Voyager 2 proton data, available from 1977 onwards, is useful for comparison, since it represents the cosmic ray flux near or at Neptune for some of the time period of interest. However, because of the variable lag of up to 4 months between the time series of neutron monitor data and Voyager 2 data, depending on the position of Voyager 2, we have chosen to focus on data from the Oulu neutron monitor, which has been in continuous operation since the 1960s (ref. 32).

### Oulu

The Oulu neutron monitor detects neutrons generated by primary GCR decaying in the atmosphere. Integrated over the atmospheric column, neutron monitor data is a reasonable proxy for cosmic ray ionization in Earth's atmosphere (although not necessarily in the lower troposphere^15^). As the physics of atmospheric ionization is fundamentally similar at Neptune and on Earth^11^, we use the Oulu data as a source of long-term cosmic ray data. The Oulu neutron monitor is essentially unchanged since installation in the 1960s, but there is an ‘efficiency' factor applied to the data to include the effects of changes in hardware and software (as well as the routinely applied atmospheric pressure correction). The data are fully explained at http://cosmicrays.oulu.fi/ and in ref. 32. Our analysis uses 1-day averages, although 5-min resolution data is available from 1968.

### Voyager

The Voyager 2 proton data (energy>70 MeV) was obtained by the LECP instrument^22^, although time series of >70 MeV ions and high-energy alphas and protons from the Cosmic Ray Subsystem instrument^33^ are similar and could equally have been used. Data (available and described in detail at http://sd-www.jhuapl.edu/VOYAGER/) are sampled at 1 s intervals, but recorded as daily average count rates, with the standard deviations reported. The median standard deviation of these was 0.003 s^−1^ (with an interquartile range of 0.002–0.004 s^−1^) compared with a median count rate of 0.072 s^−1^ that is, a fractional standard deviation of ±4%.

### Lyman-alpha ultraviolet data

The Lyman-alpha data is a composite time series of the solar hydrogen 121.57 nm emission line, and represents ultraviolet emissions from the entire solar disc^34^. It is generated from a combination of satellite measurements and models extending back to 1947, available at http://lasp.colorado.edu/lisird/lya/. For the time period relevant to this paper, the data is taken from the Atmospheric Explorer-E (1977–1980), Solar Mesospheric Explorer SME (1981–1989), the Upper Atmosphere Research Satellite (UARS) SOLSTICE instrument (1991–2000), Solar Radiation and Climate Experiment (SORCE) SOLSTICE instrument (2003–2010) and Solar Dynamics Observatory (SDO) EVE (2010–2015). The times where no satellite data is available (1972–1977 and 2001–2003) are filled in by model calculations^35^. Detailed discussion of this data set is outside the scope of this paper^35^, but the uncertainty is estimated to be ±10%.

### Spectral analysis

The periodograms in Fig. 3 were all generated from data selected using an 8-year moving window having a 0.5 cosine bell taper, with steps of 0.5 years between successive data window evaluations. The data were first de-trended to obtain magnitude fluctuations using a loess fit. (The fluctuations were demonstrated to be insensitive to the choice of detrend used in *Neptune magnitude data* above). The selected data window was cosine tapered (tapering factor 0.5), to reduce the truncation effects of the short data windows. The Lomb–Scargle^36^,^37^ approach for irregularly spaced data was used, with the code^38^ implemented in R. In the case of irregularly spaced data, the minimum detectable period is 2s, where s is the minimum sampling period of the data set. In the Neptune data the minimum sampling interval is 0.7 years, giving a minimum detectable periodicity of 1.4 years (refs 39, 40).

The 1.5–1.9-year periodicity described in the GCR data in the ‘Results' section was independently confirmed to occur in the 1980s by an additional analysis of the daily GCR data. This analysis used a phase-preserving 1.55–1.81-year Lanczos bandpass filter^41^ of half-length 8 years, with missing values addressed by multiple bootstrapped realizations^14^. Computer code is available on request.

### Droplet nucleation onto ions in Neptune's atmosphere

The saturation ratio *S* required for ions to grow into ultrafine droplets by condensation can be determined using the Thomson equation (2), which describes the equilibrium saturation ratio needed for ion-induced nucleation to become energetically favourable^11^. In equation 2 *r* is radius, *ρ* fluid density, *M* the mass of the condensing molecule, *q* charge, *γ*_*T*_ the surface tension, *k*_B_ Boltzmann's constant, *T* temperature, *ɛ*_*o*_ the permittivity of free space (all in SI units) and *ɛ*_*r*_ relative permittivity:


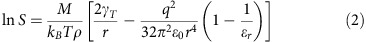


**Figure 7:**
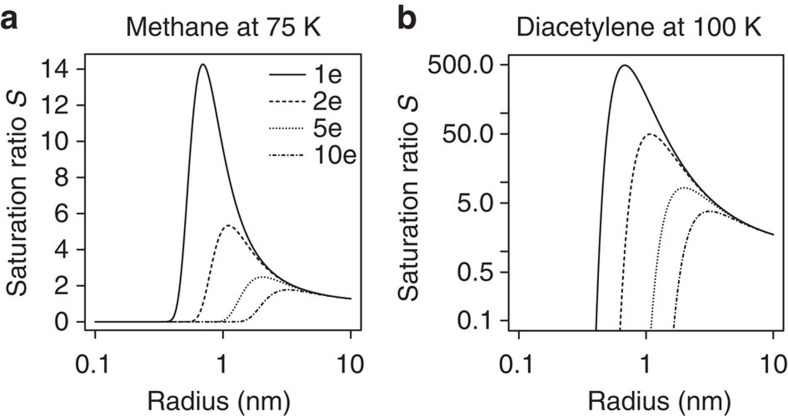
Saturation ratios required for condensation onto ions. For (**a**) methane at 75 K and (**b**) diacetylene (butadiyne) at 100 K.The critical saturation ratio required is reduced if the ions are multiply charged, with calculations given for 1, 2 and 5 elementary charges *e*.

A similar approach was taken to calculate the saturation ratio at which ion-induced nucleation could occur on Neptune^9^. Here, we apply the Thomson equation for methane and diacetylene (butadiyne), two species thought likely to form droplets through ion-induced nucleation at the pressures and temperatures appropriate for Neptune^9^. Diacetylene nucleates onto singly charged ions of critical radius 1 nm at a saturation ratio of ∼500, whereas methane needs a saturation ratio of ∼15 for nucleation onto ions (Fig. 7). As these large saturation ratios are expected in the cold Neptune environment^9^, condensation can occur onto freshly produced ∼1 nm cluster ions. Figure 7 also shows that ion-induced nucleation occurs more easily at lower saturation ratios on multiply charged large ions. For a typical charge of 2e on 10nm particles in Neptune's atmosphere, arising from asymmetry in positive ion and free electron mobilities (ref. 25), estimates of the saturation ratio required for nucleation are ∼100 (diacetylene), and ∼5 (methane), consistent with the ‘relatively efficient' ion-induced nucleation predicted in ref. 9.

### Droplet growth by condensation

We now estimate the rate of droplet growth for methane condensation at 75 K and 1,100 hPa on Neptune with methane saturation ratio of 2.7 in a hydrogen atmosphere^9^. (There is not enough data available to carry out the calculation for diacetylene).

Following ref. 24, the rate of growth of a droplet of radius *r* is given by


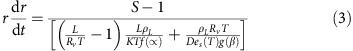


where, *S* is the saturation ratio, *T* is the temperature, *L* is the latent heat of vapourization, *R*_v_ is the gas constant for the condensing species, *D* is the diffusion coefficient for the condensing species, *K* is the thermal conductivity, *ρ*_*v*_ is the vapour density and *e*_s_*(T)* is the saturation vapour pressure. Two normalization factors, *f(α)* and *g(β)*, are also defined^24^ where,


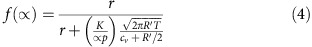


with *p* the pressure and *R'* the gas constant of the background gas, and α is 1. The second normalization factor is given by


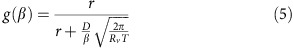


where, 0.02<*β<*0.04 and is taken here to be 0.04. The terms used in the calculation are listed in Table 3.

Inserting values from Table 3, we find that the estimated droplet growth rate is of order 4 nm s^−1^, which is insensitive to both the initial radius, and the radius as the droplet grows. This insensitivity to radius arises from the 1/*r* term in equation 3 being compensated by the *r* terms in equations 4 and 5.

## Additional information

**How to cite this article:** Aplin, K. L. & Harrison, R. G. Determining solar effects in Neptune's atmosphere. *Nat. Commun.* 7:11976 doi: 10.1038/ncomms11976 (2016).

## Supplementary Material

Supplementary Information Supplementary Figure 1

## Figures and Tables

**Table 1:** Summary of multiple regression analysis.

Case	Physical interpretation	Coefficients in equation (1)	Adjusted coefficient of determination (*R*^2^) and statistical significance *P* value	
			*y*(551 nm)	*b*(472 nm)
1	GCR only (ion-particle attachment limited)	*κ*=0, *μ*=0; *λ* free to vary	0.02	0.13 (*P*<*0.01*)
2	√GCR only (ion–ion/ion–electron recombination limited)	*κ*=0, *λ*=0; *μ* free to vary	0.02	0.13 (*P*<*0.01*)
3	GCR+√GCR	*κ*=0, *λ* and *μ* free to vary	0.03	0.12 (*P*<*0.05*)
4	Ultraviolet only	*λ*=0, *μ*=0; *κ* free to vary	0.08 (*P*<*0.05*)	0.20 (*P*<*0.01*)
5	Ultraviolet+GCR	*μ*=0; *κ* and *λ* free to vary	0.08 (*P*<*0.05*)	0.18 (*P*<*0.1*)
6	Ultraviolet+√GCR	*λ*=0; *κ* and *μ* free to vary	0.07 (*P*<*0.1*)	0.18 (*P*<*0.1*)
7	Ultraviolet+GCR+√GCR	*κ*, *λ* and *μ* free to vary	0.14 (*P*<*0.05*)	0.19 (*P*<*0.05*)

GCR, galactic cosmic ray.

Fits are weighted according to the errors on the measurements^3^. Statistical significances of the fits are indicated where better than 90% (*P*<0.1). The adjusted coefficient of determination (*R*^2^) gives the fraction of the variance explained by the fit, whilst accounting for the different number of variables in each fit.

**Table 2:** Kolmogorov–Smirnov test results (*P* values) for Neptune magnitude fluctuations de-trended with different techniques.

	Lowess	Loess	Quadratic
Lowess		**0.436**	**0.292**
Loess	**0.292**		0.035
Quadratic	**0.064**	0.009	

If *P*>0.05 the data do not statistically differ from each other, indicated in bold. The *b* 472 nm data are shown bottom left and the *y* 551 nm data are shown top right.

**Table 3:** Quantities used to estimate growth rate of methane droplets in hydrogen at ∼100 K.

Quantity and description	Value used (SI units)	Comment
*c*_v_ specific heat of background gas at constant volume	6.236 × 10^3^ J kg^−1^ K^−1^	Assumes no modes are excited at these temperatures, so 3/2*k*_B_ per mole (where *k*_B_ is the Boltzmann constant) (for example, see ref. 42)
*D* diffusion coefficient	3 × 10^−5^ m^2^s^−1^	at 90 K (methane diffusing in methane)^43^
*e*_*s*_*(T)* saturation vapour pressure	1.03 Pa	Calculated at 75 K ref. 9
*K* thermal conductivity	0.509 W m K^−1^	at 73.2 K and 1,013 hPa ref. 44
*L* latent heat of vapourization	5.1 × 10^5^ J kg^−1^	at 110 K ref. 45
*R'* gas constant (background)	4.124 × 10^3^ J kg^−1^ K^−1^	Calculated from universal gas constant and molecular mass
*R*_v_ gas constant (condensing species)	5.183 × 10^2^ J kg^−1^ K^−1^	
*r* radius	Initial value 1 nm	
*P* pressure	1.1 × 10^5^ Pa	Conditions defined in ref. 9^9^
*S* supersaturation	2.7	
*T* temperature	75 K	
α-coefficient	1	From ref. 24
β-coefficient	0.04	
*ρ*_v_ density of condensing species	471 kg m^−3^	Calculated at 75 K ref. 9

## References

[b1] Lockwood G. W. & Thompson D. T. Photometric variability of Neptune, 1972–2000. Icarus 156, 37–51 (2002).

[b2] Lockwood G. W. & Jerzykiewycz M. Photometric variability of Uranus and Neptune, 1950–2004. Icarus 180, 442–452 (2006).

[b3] Lockwood G. W. Photometry of Uranus and Neptune, 1972–2014. Available at http://www2.lowell.edu/users/wes/index.htm (accessed on 26 June 2015).

[b4] Fletcher L. N., Drossart P., Burgdorf M., Orton G. S. & Encrenaz T. Neptune's atmospheric composition from AKARI infrared spectroscopy. Astron. Astrophys. 514, A17 (2010).

[b5] Karkoschka E. Neptune's cloud and haze variations 1994–2008 from 500 HST–WFPC2 images. Icarus 2015, 759–773 (2011).

[b6] Baines K. H. & Smith W. H. The atmospheric structure and dynamical properties of Neptune derived from ground-based and IUE spectrophotometry. Icarus 85, 65–108 (1990).

[b7] Lockwood G. W. & Thompson D. T. Long-term brightness variations of Neptune and the solar cycle modulation of its albedo. Science 234, 1543–1545 (1986). 17816506 10.1126/science.234.4783.1543

[b8] Moses J. I., Allen M. & Yung Y. L. Neptune's visual albedo variations over a solar cycle: a pre-Voyager look at ion-induced nucleation and cloud formation in Neptune's troposphere. Geophys. Res. Lett. 16, 1489–1492 (1989).

[b9] Moses J. I., Allen M. & Yung Y. L. Hydrocarbon nucleation and aerosol formation in Neptune's atmosphere. Icarus 99, 318–346 (1992). 11538166 10.1016/0019-1035(92)90149-2

[b10] Wilson C. T. R. On a method of making visible the paths of ionising particles through a gas. Proc. Roy. Soc. A 85, 285–288 (1911).

[b11] Aplin K. L. Atmospheric electrification in the Solar System. Surv. Geophys. 27, 63–108 (2006).

[b12] Mironova I. A. . Energetic particle influence on the Earth's atmosphere. Space Sci. Rev. 194, 1–9 (2015).

[b13] Valdés-Galicia J. F., Pérez-Enríquez R. & Otaoloa J. A. The cosmic-ray 1.68 year variation: a clue to understand the nature of the solar cycle? Solar Phys. 167, 409–417 (1996).

[b14] Harrison R. G. Discrimination between cosmic ray and solar irradiance effects on clouds, and evidence for geophysical modulation of cloud thickness. Proc. Roy. Soc. A 464, 2575–2590 (2008).

[b15] Harrison R. G., Nicoll K. A. & Aplin K. L. Vertical profile measurements of lower troposphere ionisation. J. Atmos. Solar. Terr. Phys. 119, 203–210 (2014).

[b16] Vaughan S. Scientific Inference: Learning from Data Cambridge University Press (2013).

[b17] Pryor W. R. & Hord C. W. A study of photopolarimeter system UV absorption data on Jupiter, Saturn, Uranus, and Neptune: implications for auroral haze formation. Icarus 91, 161–172 (1991).

[b18] Kato C., Munakata K., Yasue S., Inoue K. & McDonald F. B. A 1.7-year quasi-periodicity in cosmic ray intensity variation observed in the outer heliosphere. J. Geophys. Res. 108, 1367 (2003).

[b19] Rouillard A. & Lockwood M. Oscillations in the open solar magnetic flux with a period of 1.68 years: imprint on galactic cosmic rays and implications for heliospheric shielding. Ann. Geophys. 22, 4381–4395 (2004).

[b20] Kane R. P. Short-term periodicities in solar indices. Solar Phys. 227, 155–175 (2005).

[b21] Harrison R. G. & Märcz F. Heliospheric timescale identified in surface atmospheric electricity. Geophys. Res. Lett. 34, L23816 (2007).

[b22] Krimigis S. M. . The Low Energy Charged Particle (LECP) experiment on the Voyager spacecraft. Space Sci. Rev. 21, 329–354 (1977).

[b23] Stone E. C. & Miner E. D. The Voyager 2 encounter with the Neptune system. Science 246, 1417–1421 (1989). 17755996 10.1126/science.246.4936.1417

[b24] Rogers R. R. & Yau M. L. A Short Course in Cloud Physics 3rd edn Pergamon Press (1989).

[b25] Clement C. F. & Harrison R. G. The charging of radioactive aerosols. J. Aerosol Sci. 23, 481–504 (1992).

[b26] Stoker C. R. & Toon O. Moist convection on Neptune. Geophys. Res. Lett. 16, 929–932 (1989).

[b27] Hammel H. B. & Lockwood G. W. Long-term atmospheric variability on Uranus and Neptune. Icarus 186, 291–301 (2007).

[b28] Cleveland W. S. LOWESS: a program for smoothing scatterplots by robust locally weighted regression. Am. Stat. 35, 54 (1981).

[b29] Cleveland W. S., Grosse E. & Shyu W. M. Local regression models. In: Statistical Models in S eds Chambers J. M., Hastie T. J. Wadsworth & Brooks/Cole (1992).

[b30] Crawley M. J. Statistics: an introduction using R Wiley (2005).

[b31] Nakamura K. . (Particle Data Group) Review of particle physics (section 24: Cosmic rays). J. Phys. G: Nucl. Part. Phys. 37, 075021 (2010).

[b32] Usoskin I. G., Mursula K., Kangas J. & Gvozdevsky B. On-line database of cosmic ray intensities. Proc. Int. Cosmic Ray Conf. 2001, 2–4 (2001).

[b33] Stone E. C. . Cosmic ray investigation for the Voyager missions: energetic particle studies in the outer heliosphere—and beyond. Space Sci. Rev. 21, 355 (1977).

[b34] Lean J. L. & Skumanich A. Variability of the Lyman alpha flux with solar activity. J. Geophys. Res. 88, 5751–5759 (1983).

[b35] Woods T. N., Tobiska W. K., Rottman G. J. & Worden J. R. Improved solar Lyman alpha irradiance modelling from 1947 through 1999 based on UARS observations. J. Geophys. Res. 105, 27195–27215 (2000).

[b36] Lomb N. R. Least squares frequency analysis of unequally spaced data. Astrophys. Space Sci. 39, 447–462 (1976).

[b37] Scargle J. D. Studies in astronomical time series analysis. II—Statistical aspects of spectral analysis of unevenly spaced data. Ap. J. 263, 835–853 (1982).

[b38] Press W. H. & Rybicki G. B. Fast algorithm for spectral analysis of unevenly sampled data. Ap. J. 338, 277–280 (1989).

[b39] Eyer L. & Bartholdi P. Variable stars: which Nyquist frequency? Astron. Astrophys. Supp. Ser. 135, 1 (1999).

[b40] Koen C. The Nyquist frequency for irregularly spaced timeseries: a calculation formula. Mon. Not. R. Astron. Soc. 371, 1390–1394 (2006).

[b41] Duchon C. E. Lanczos filtering in one and two dimensions. J. Appl. Meteor 18, 1016–1022 (1979).

[b42] Blundell S. J. & Blundell K. M. Concepts in Thermal Physics Oxford University Press (2006).

[b43] Winn E. B. The temperature dependence of the self-diffusion coefficients of argon, neon, nitrogen, oxygen, carbon dioxide and methane. Phys. Rev. 80, 1024 (1950).

[b44] Kaye G. W. C. & Laby T. H. Tables of physical and chemical constants 16th edn Longman (1995).

[b45] Lide D. R. (ed). CRC handbook of chemistry and physics 76th edn CRC Press (1995).

