# Supplementary material for: Determining solar effects in Neptune's atmosphere
Source: Nat Commun. 2016 Jul 15;7:11976. doi: 10.1038/ncomms11976 (PMC4947159; doi:10.1038/ncomms11976)
Supplement: Supplementary Information — Supplementary Figure 1 [file ncomms11976-s1.pdf]

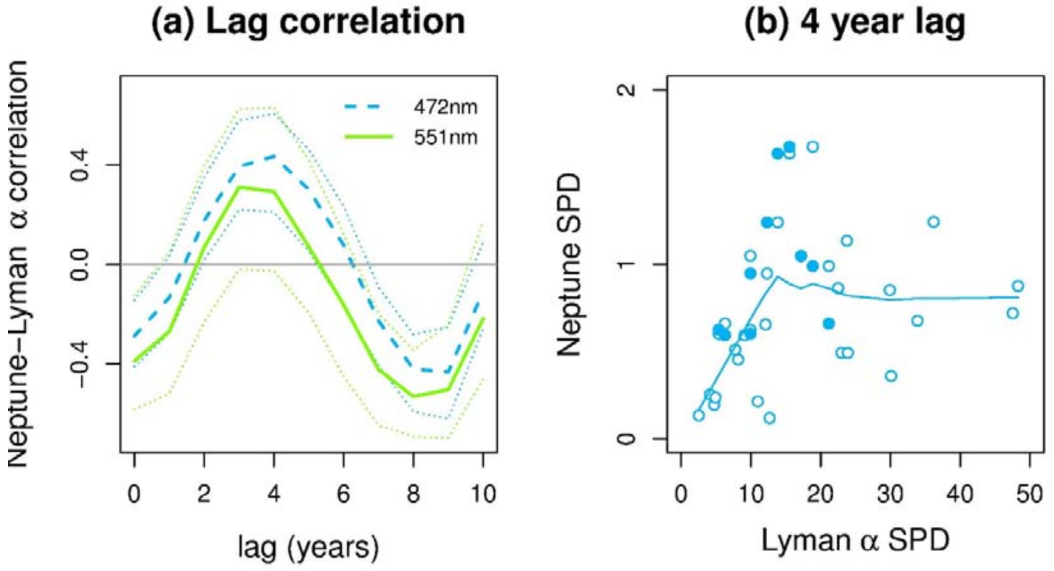

Supplementary Figure 1

Relationships between 1.5-1.9 year spectral power densities for Neptune and ultra-violet radiation.

(a) Correlation between mean normalised spectral power density (SPD) for periodicities between 1.5 and 1.9 years in Neptune's atmosphere against the same periodicity in Lyman alpha (UV) radiation, for Neptune lagging UV by 0-10 years. Dotted lines mark 95% confidence limits from multiple (10000) realisations of the SPDs calculated with the uncertainties in the magnitude fluctuations. (b) Average Neptune 472nm SPD against UV SPD data values for the 1.5-1.9 year periodicity, with Neptune lagging UV by 4 years. (This difference in lag compared to figure 5 is unlikely to be important, because a 1.5-1.9 year periodicity calculated from the quasi-annually sampled Neptune data gives the lag determination at best 2 year resolution.) The filled circles are from 1980-1989, when the 1.5-1.9 year periodicity in GCR was particularly strong, and the rest of the data are open circles. A lowess fit to all the data points is also shown (solid line).
